# Electricity in the Diseases of Women

**Published:** 1890-09

**Authors:** 


ELECTRICITY IN THE DISEASES OF WOMEN. By G. BETTON MASSEY, M. D., Physician to the Gynecological Department of Howard University, Late Electro-therapeutist to the Philadelphia Orthopedic Hospital, etc. Second edition; revised and enlarged. Philadelphia and London: F. A. Davis, Publisher. 1890.

The first edition of this little work appeared in 1889, and in less than a year the author is called upon to prepare a second. This shows how a love of the marvelous and unknown leads not alone the masses, but the profession, that ought to be more conservative, into curious demands that border on the extravagant if not ridiculous.

We devoted considerable space in the JOURNAL to an analytical review of the former edition, so we need not trench upon the valuable time of our readers with this subject now, particularly as nothing has since been put forth to demonstrate that we committed any error of judgment in our criticisms, pro or con, passed at that time.

In this edition "it was found necessary to rewrite the greater portion of chapters xi., xii., xiv. and xvi., and add new chapters on subinvolution and chronic inflammatory diseases of appendages," because "so considerable has been the progress of definite knowledge in the art."

Graphic representations of the law of Ohm and of the laws of current diffusion have been added to the appendix for the convenience of students. The book is handsomely printed, and bound in the same style as the former edition.

